# Complications of Intra-Arterial tPA for Iatrogenic Branch Retinal Artery Occlusion: A Case Report through Multimodal Imaging and Literature Review

**DOI:** 10.3390/medicina57090963

**Published:** 2021-09-13

**Authors:** Katherine Dalzotto, Paige Richards, Tyler D. Boulter, Marilyn Kay, Mihai Mititelu

**Affiliations:** Department of Ophthalmology and Visual Sciences, University of Wisconsin School of Medicine and Public Health, Madison, WI 53705, USA; prichards@uwhealth.org (P.R.); tboulter@uwhealth.org (T.D.B.); mkay3@wisc.edu (M.K.); mititelu@wisc.edu (M.M.)

**Keywords:** retinal artery occlusion, tPA, spectral domain optical coherence tomography, optical coherence tomography angiography

## Abstract

*Background and Objectives*: To document, through multimodal imaging, the post-procedural clinical course and visual outcome of a patient who received intra-arterial tissue plasminogen activator (tPA) for acute iatrogenic branch retinal artery occlusion (BRAO), and to review the literature and guidelines regarding the use of tPA for retinal arterial occlusions. *Methods*: A 28-year-old female patient who sustained an iatrogenic BRAO and subsequently received intra-arterial tPA was followed through her post-interventional course of 3 months with serial exams and multimodal imaging, including color fundus photography, visual field testing, spectral domain optical coherence tomography (SD-OCT), and OCT angiography (OCT-A). *Results*: A patient with history of left internal cerebral artery (ICA) aneurysm and baseline visual acuity (VA) of 20/20 developed an acutely symptomatic BRAO after undergoing a neuroendovascular procedure and was acutely treated with tPA through the left ophthalmic artery. At two weeks follow-up, a central posterior pole hemorrhage was noted although VA was preserved. A superior altitudinal defect was shown on automated perimetry. VA dropped to 20/50 at 7 weeks follow-up and hyperreflective material deep to the attachment between the posterior hyaloid and the internal limiting membrane (ILM) consistent with hemorrhage was noted on SD-OCT. At 11 weeks follow-up, VA returned to 20/20, SD-OCT revealed a membrane bridging the foveal depression, OCT-A showed decreased vascularity in the inferior macula, and the visual field defect was stable by automated perimetry. *Conclusions*: Intraocular hemorrhage is a possible complication of intra-arterial tPA administration for BRAO, and a careful analysis of risks, benefits, and goals of this procedure must be considered by both provider and patient before such intervention.

## 1. Introduction

Central retinal artery occlusion (CRAO) and branch retinal artery occlusion (BRAO) are vascular occlusive events resulting in ischemia of retinal tissue. Nonhuman primate studies have demonstrated that there is severe injury to retinal tissue after just 97–105 min of ischemia [1], and that window may be extended to 240 min under conditions of chronic hypertension and atherosclerosis, suggesting a development of tolerance over time [2].

Intravenous and intra-arterial tissue plasminogen activator (tPA) are well-established treatments for ischemic cerebrovascular events and have well-defined guidelines for use to maximize safety and minimize risk of complications with various guidelines from the neurology and neurosurgery literature. CRAO (and BRAO) fit the definition of acute ischemic stroke as set forth by the American Heart Association/American Stroke Association [3]. Likewise, a penumbra of retinal tissue has been demonstrated which parallels the penumbra of cerebral tissue in acute ischemic stroke [4].

Before the advent of tissue plasminogen activator (tPA) use in retinal arterial occlusions, various interventions (ranging from ocular massage to the use of intraocular pressure (IOP)-lowering medications to anterior chamber paracentesis) had been described as potential interventions for these ischemic events. A meta-analysis by Schrag et al. has shown not only that these treatments are ineffective, but they may confer a higher risk of complications and worse visual outcomes [5].

Although most emboli in retinal artery occlusion are cholesterol- or calcium-based, they may still have some fibrinous content, making them somewhat amenable to thrombolysis [6].

There are only two randomized control trials evaluating the role of tPA in the management of retinal artery occlusion. The European Assessment Group for Lysis in the Eye (EAGLE) study evaluated the role of intra-arterial tPA and found no significant difference in visual acuity recovery between treatment (42 patients) and control (40 patients) groups, and more importantly was aborted due to safety concerns and lack of efficacy of the experimental treatment [7]. The study by Chen et al. included 16 participants (equally divided between treatment group and controls) and found a general trend towards benefit of intravenous tPA on visual acuity recovery [8].

The 2015 meta-analysis by Schrag et al. examined studies dating back to 1966 (with a total of 144 patients receiving thrombolysis) and showed promise that intravenous thrombolysis early in the disease course (within 4.5 h of onset) led to a rate of visual acuity recovery of 50% [5].

Additional support for treatment of CRAO as acute ischemic stroke was published by MacGrory et al. in the neurology literature in 2019, adding 3 more case-series (with a total of 61 new patients) to the growing literature [9]. They concluded in favor of thrombolysis within 4.5 h of symptom onset, and acknowledged the need for further randomized trials to support this practice.

A meta-analysis by Dumitrascu et al. from the neuro-ophthalmology literature included the 3 recent series from MacGrory et al.’s report above, the 2011 trial by Chen et al., two studies included in the earlier Schrag et al. meta-analysis, as well as an older two-case series [5,8,9,10]. It found a general trend towards better visual acuity outcome when patients had received some form of tPA within recommended time cutoffs (4.5 h for intravenous tPA, 6 h for intra-arterial tPA).

Although parallels exist between cerebral ischemia and retinal ischemia, the use of tPA for treatment of the latter remains controversial and there are differing approaches among the ophthalmology and neurology communities. Aided by multimodal imaging, we aim to describe the post-procedural course and recovery of a patient with iatrogenic BRAO who received intra-arterial tPA and developed a visually significant intraocular hemorrhage.

## 2. Case Report

**Figure 1:**
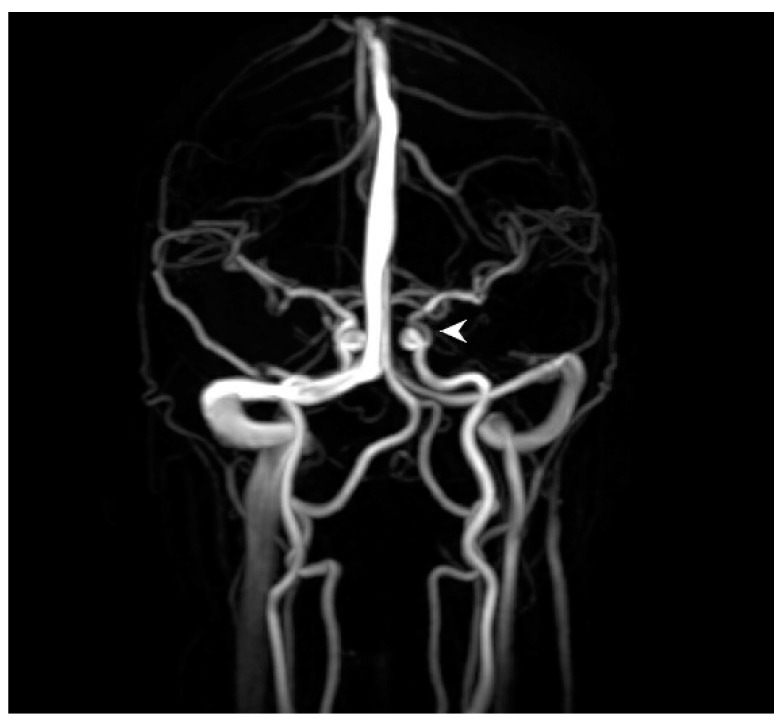
Brain magnetic resonance angiography (MRA) immediately prior to ophthalmic examination shows anticipated changes from the recent revascularization procedure with persistent filling of the 2 mm supraclinoid segment aneurysm (arrowhead), without other abnormalities of the major intracranial vasculature.

A 28-year-old woman with a history of migraines, prior tobacco use, narcolepsy, basilar artery occlusion (thought to be secondary to chiropractic manipulation) with resulting internuclear ophthalmoplegia without persistent defects, and asymptomatic left internal carotid artery (ICA) paraophthalmic aneurysm underwent a flow diversion procedure for the ICA aneurysm. She reported “I can’t see out of the top of my left eye” upon awakening in the postoperative care area. Emergency magnetic resonance imaging (MRI) showed no evidence of acute infarct, hemorrhage, or other intracranial abnormality. Magnetic resonance angiography/venography (MRA/MRV) revealed postprocedural changes of the left ICA but no other abnormalities of the intracranial vasculature (Figure 1).

**Figure 2:**
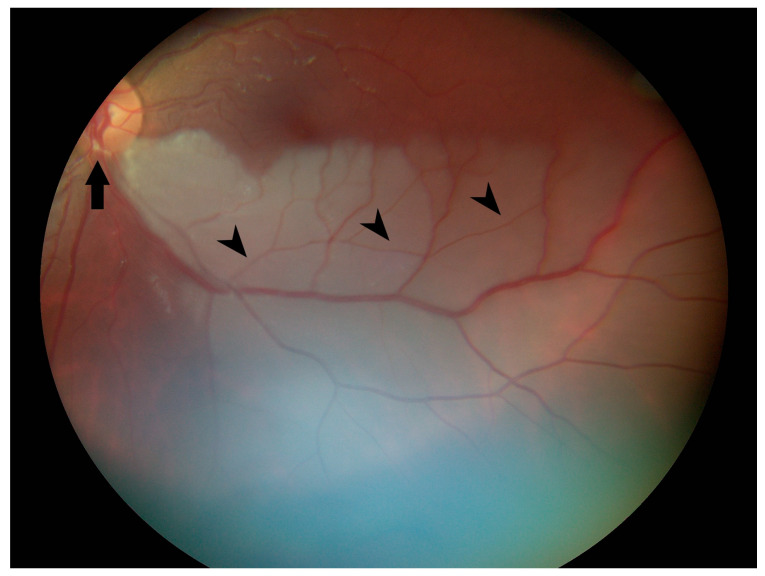
Color fundus photograph of the left eye at presentation shows retinal whitening of the inferior fundus consistent with diagnosis of branch retinal artery occlusion. Note the yellow refractile body within the inferior arterial arcade at the disc margin (arrow) and attenuation of peripheral arterioles (arrowheads).

Ophthalmology was consulted after the vision changes did not resolve with 1000 mg of acetaminophen. Visual acuity was 20/20 in the right eye and 20/30 in the left eye. Pupils were equal, round, and reactive to light without relative afferent pupillary defect. Intraocular pressure was 10 mmHg in both eyes. Confrontational visual fields (via counting fingers) were normal in the right eye and revealed a superonasal defect in the left eye. Funduscopic exam of the right eye was normal, but in the left eye, there was frank whitening of the inferior half of the retina and a visible yellow refractile body within the inferior arterial arcade at the disc margin. Branches of the retinal artery downstream from this plaque were narrowed/attenuated (Figure 2). She was diagnosed with branch retinal artery occlusion (BRAO).

The neurosurgery service felt the risk-benefit ratio of acute intervention with tPA was in this patient’s favor given her young age, her relative state of health, and their previous experience with similar clinical scenarios. The etiology of the embolus was thought to be due to dislodging of part of an early atherosclerotic plaque during the previous neuroendovascular procedure. Six hours after the patient’s initial complaint of visual field defect (9.5 h from last known normal), intra-arterial administration of 10.8 mg of tPA into the left ophthalmic artery was performed by the neurosurgery team in hopes of re-perfusing the retinal tissue in the penumbra of the initial ischemic event.

Upon follow-up examination at bedside the next day, visual field and funduscopic exam findings were objectively unchanged (including the appearance of the embolus), although the patient described subjective improvement in the visual field defect. She was otherwise neurologically well.

**Figure 3:**
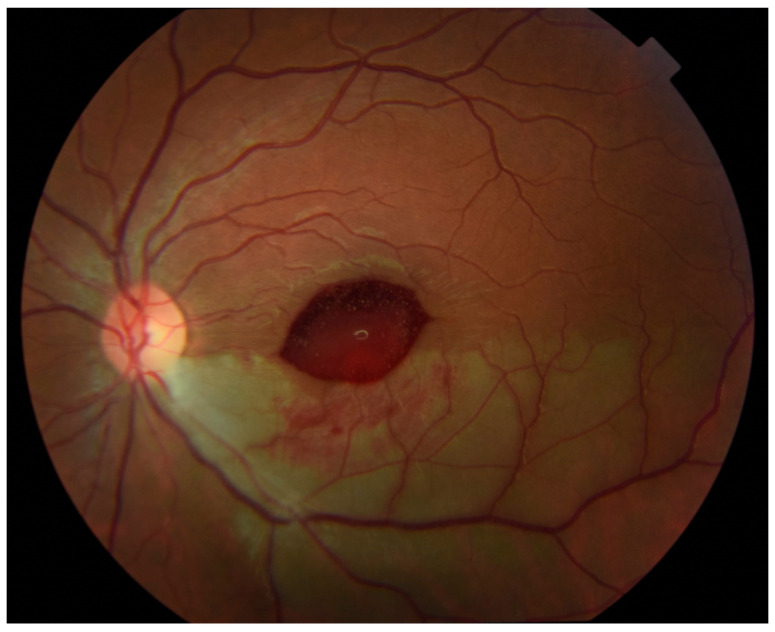
Color fundus photograph of the left eye at follow-up 2 weeks after administration of tissue plasminogen activator (tPA) shows new ellipsoid-shaped hemorrhage blocking the central macula and slightly improved inferior retinal whitening.

Outpatient follow-up two weeks later revealed visual acuity in the left eye of 20/20 eccentrically. Humphrey visual field 24-2 (Carl Zeiss-Humphrey Systems, Dublin, CA, USA) revealed a superior altitudinal defect, denser nasally. Color fundus photographs showed an ellipsoid area of hemorrhage obscuring the central macula (Figure 3). The patient had not noticed any subjective decrease in her vision so it is unknown exactly when in the period between 24 h and 2 weeks after intra-arterial tPA that this hemorrhage developed.

**Figure 4:**
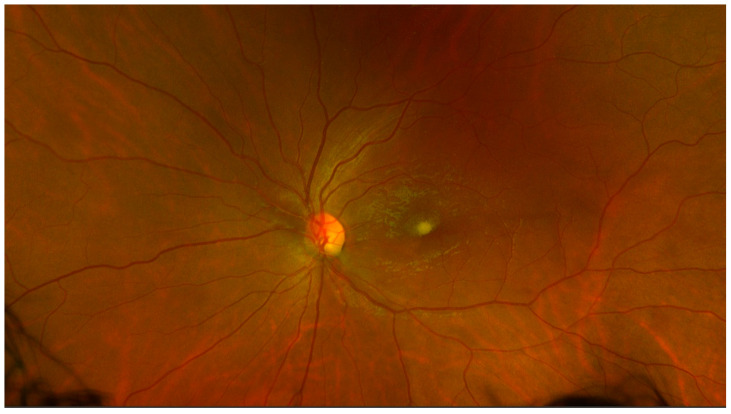
Color fundus photograph of the left eye at 7 weeks follow-up shows significant improvement of inferior retinal whitening and significant resorption of central macular hemorrhage. Note the yellowish superficial deposit over the fovea, consistent with residual dehemoglobinized blood.

**Figure 5:**
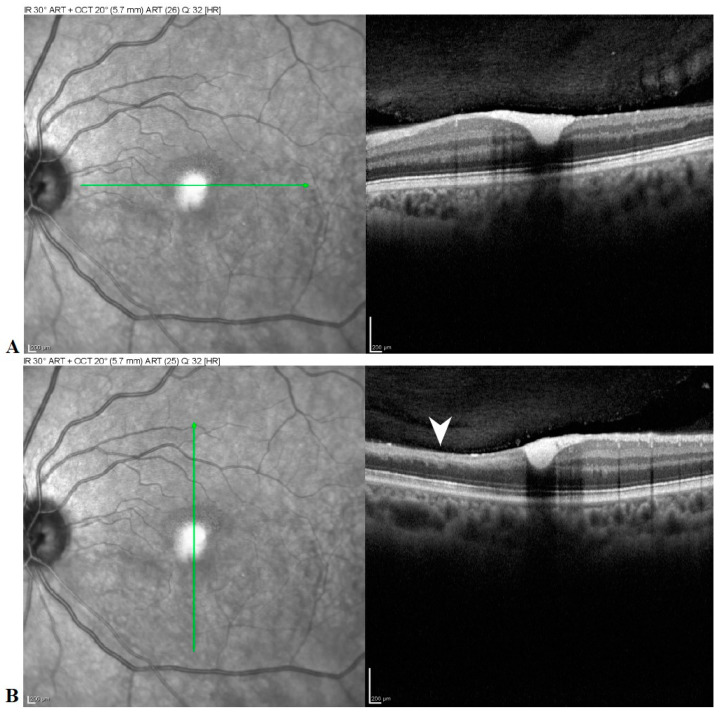
Horizontal (**A**) and vertical (**B**) spectral domain OCT B-scans show hyperreflective material deep to the attachment between the posterior hyaloid and the internal limiting membrane (ILM) and filling the foveal depression suggestive of residual hemorrhage. Note the thinning of the inner retinal layers inferiorly from recent branch retinal artery occlusion (arrowhead).

At 7 weeks after the onset of the BRAO, visual acuity in the left eye had dropped to 20/50 and color fundus photos revealed a yellowish discoloration over the fovea (Figure 4), consistent with dehemoglobinized blood. Humphrey visual fields revealed a stable defect. SD-OCT (Spectralis; Heidelberg Engineering, Heidelberg, Germany) showed inner retina hyperreflective material beneath the internal limiting membrane (ILM) consistent with resolving hemorrhage, along with expected thinning and loss of inner retinal layers in the distribution of the BRAO (Figure 5).

**Figure 6:**
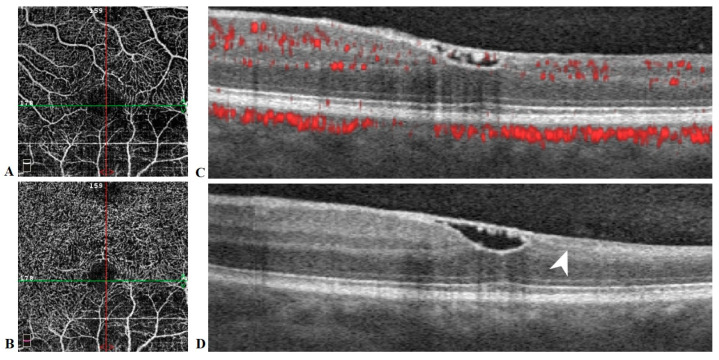
Optical coherence tomography angiography (OCTA) en face images (**A**,**B**) and spectral domain OCT B-scans (**C**,**D**) of the left eye at 11 weeks follow-up. Decreased vessel density of the superficial (**A**) and deep (**B**) retinal capillary plexuses is noted inferiorly in the area of arterial occlusion. A contiguous membrane with mild residual hyper-reflective hemorrhage underneath traverses the foveal depression (**C**,**D**). Vertical (**D**) OCT shows significant thinning of inner retinal layers of the inferior macula (arrowhead).

At 11-week follow-up, visual acuity in the left eye had returned to 20/20. Funduscopic exam was significant for complete resorption of blood and a residual wrinkly yellow sheen over the central macula. Humphrey visual fields remained stable. SD-OCT revealed a bridging membrane over the foveal depression. OCT-A (AngioVue; Optovue Inc., Fremont, CA, USA) was limited by artifact but suggested decreased retinal vascular bed density in the inferior macula (Figure 6).

## 3. Discussion

We report a novel case of iatrogenic BRAO which was treated with intra-arterial tPA and later developed intraocular hemorrhage, documented by serial exams and multimodal imaging. This case highlights the development of visually significant tPA side-effects, however as reviewed below, the systemic complications of tPA treatment also remain of major interest due to their risk of mortality.

Two randomized control trials have been conducted to evaluate the efficacy and safety of tPA in nonarteritic CRAO, specifically the EAGLE study using intra-arterial tPA and a similar trial published by Chen et al. [7,8]. The EAGLE study showed no difference between the tPA group and control group in terms of visual outcome but did show a significantly higher rate of complications (one cerebral hemorrhage, one cerebellar hemorrhage, and two minor non-ocular hemorrhages) in the tPA group which contributed to the early termination of the trial [7]. It bears noting that in the EAGLE trial, patients did receive tPA outside the now-accepted window of 4.5 h from the last known normal (as it pertains to the treatment of acute stroke), which may have contributed to the higher complication rate.

The 2011 trial by Chen et al. did suggest a benefit of early tPA administration (within 6 h of onset) but was limited by small sample size of 8 patients in the treatment group. One individual who received tPA had an intracranial hemorrhage resulting in need for hemicraniectomy, but this was later attributed to comorbid cerebral amyloid angiopathy. There were no intraocular hemorrhages in their patient cohort [8].

The meta-analysis by Schrag et al. from the neurology literature showed potential benefit of early thrombolysis, but also found 5 hemorrhages which were linked to the older thrombolytic streptokinase [5]. Four of the hemorrhages were fatal (3 intracerebral, 1 hepatic). Their subgroup analysis of tPA-only patients who received it within 4.5 h of onset revealed visual recovery in 8 of 13 study participants and no hemorrhagic complications. There was no comment regarding the development of intraocular hemorrhages [5].

Out of the 61 patients analyzed by MacGrory et al., there were a total of 6 complications: one symptomatic intracranial hemorrhage (in a case where the standard anticoagulation protocol was broken), two asymptomatic intracranial hemorrhages, one case of hematuria, one case of angioedema, and one abdominal aortic aneurysm bleed [9].

In the additional studies included in the 2020 meta-analysis by Dumitrascu et al., none of the 42 patients had any notable complications such as symptomatic intracranial or ocular hemorrhage [10]. The authors concluded with the following recommendation, echoing the sentiment of MacGrory et al. one year earlier [9]: “Nonarteritic CRAO patients should theoretically receive the same thrombolytic therapies, in the same time window, as patients with acute cerebral ischemia”.

Two studies in particular have evaluated the use of orbital sonography in the workup of CRAO. Ertl et al. established orbital sonography as a way to diagnose embolic CRAO with the “spot sign”—hypoperfusion in the central retinal artery with hyperechoic material—which had a sensitivity of 83% and a specificity of 100% for CRAO [11]. Nedelmann et al. expanded on this work and hypothesized that the spot sign reflects calcified contents of the embolus. In their prospective study of 46 patients, only those without a “spot sign” experienced clinically significant visual improvement [12].

Our report highlights differences in the approach to management of retinal arterial occlusions between the neurology and ophthalmology literatures. The ophthalmology community has remained cautious regarding the use of tPA for retinal artery occlusions, citing concerns over unproven efficacy, safety risks, and the lack of consistently improved visual outcomes from randomized controlled trials. One oft-cited reason for this hesitancy is that patients with these events often present to outpatient clinic, often outside the critical time window for safe tPA administration (as established for acute stroke). If the ophthalmology community changes practice towards routine administration of tPA for acute retinal occlusive events within the proper time window, it would be imperative to have a full understanding of the possible complications (as well as their management and prognosis) of this intervention. As many have recognized the parallels between revascularized retina and brain tissue, as well as the increased risk of intracranial bleeding with tPA, we too must recognize the potential for hemorrhagic complications affecting the retina.

Although the studies reviewed have specifically described intracranial hemorrhage after tPA administration, no studies have focused on intraocular hemorrhages as a complication of this intervention. To our knowledge, this is the first documented case of iatrogenic BRAO treated with intra-arterial tPA and complicated by the development of intraocular hemorrhage, and the first report to follow the clinical course after such an event through multimodal imaging. Although our patient underwent spontaneous resolution of hemorrhage, this complication can have more significant implications for patients who are monocular or who may require intervention to manage non-clearing intraocular hemorrhages after tPA administration.

Additionally, our patient underwent this procedure for BRAO, for which there is a paucity of reperfusion data published compared to CRAO. She was treated with tPA outside the recommended window of 4.5 h (as established for acute stroke). Moreover, this patient’s occlusive event was iatrogenic in nature, which is not well described in the published literature.

## 4. Conclusions

In order to ensure best patient outcomes, it is important to understand the proper window of treatment and to balance treatment outcomes with the nature and seriousness of the systemic and ocular side effects. Continued multidisciplinary discussion between specialties (ophthalmology, neurology, neurosurgery) managing this condition remains key if and when tPA becomes more widely used for treatment of retinal artery occlusions.

## Data Availability

No new data were created or analyzed in this study. Data sharing is not applicable to this article.
